# Addressing the Global Threat of Multidrug-Resistant Infections: The Role of Ceftazidime-Avibactam Revisited

**DOI:** 10.7759/cureus.60235

**Published:** 2024-05-13

**Authors:** Sreejith Raveendran, Deepashree R, Sujatha Shimoga Ravi Kumar, Krishna Karthik

**Affiliations:** 1 Microbiology, JSS Medical College and Hospital, JSS Academy of Higher Education and Research, Mysore, IND; 2 Clinical Microbiology, JSS Medical College and Hospital, JSS Academy of Higher Education and Research, Mysore, IND; 3 Medical Microbiology, JSS Medical College and Hospital, JSS Academy of Higher Education and Research, Mysore, IND

**Keywords:** ceftazidime-avibactam, antimicrobial resistance, gram negative bacteremia, gram negative rods, multi-drug resistant organism (mdro)

## Abstract

Background and objective

Bloodstream infections (BSIs) due to multidrug-resistant Gram-negative bacteria (MDR-GNB) pose a significant global health threat amid rising antimicrobial resistance (AMR). This study aimed to investigate the efficacy of ceftazidime-avibactam (CZA) as a therapeutic option for these infections, addressing the urgent need for novel treatments.

Materials and methods

This study was conducted over one year in the Department of Microbiology, JSS Medical College and Hospital, Mysuru, India, and employed a laboratory-based prospective design. From a total of 376 positive blood cultures, 147 multidrug-resistant (MDR) organisms were identified, and 100 were randomly selected for final analysis. Susceptibility testing via disk diffusion and minimum inhibitory concentration (MIC) determination was performed to evaluate CZA efficacy.

Results

*Klebsiella pneumoniae (K. pneumoniae) *was the predominant (78%) organism among the subsets, with varying susceptibility patterns observed across species. The overall CZA susceptibility was 45%, with significant discrepancies between disk diffusion and gold standard testing. Notably, there was limited efficacy against *Pseudomonas aeruginosa (P. aeruginosa)*

Conclusions

This study underscores the pressing need for reliable testing methods and novel treatment strategies in combating MDR infections. Further research with larger sample sizes is imperative to validate our findings and guide clinicians effectively in addressing this critical health challenge.

## Introduction

Antimicrobial resistance (AMR) is one of the major challenges in global public health, posing a significant threat to the effective treatment of infections. In recent decades, the proliferation of multidrug-resistant organisms (MDROs) has intensified, driven by the overuse of antibiotics across various sectors and a dearth of novel antibiotic development initiatives. This trend has led to the emergence of "difficult-to-treat" infections, with high morbidity and mortality rates. Consequently, strategies such as the "bad bugs, no drugs" campaign and the 10 x '20 Initiative have underscored the urgent need for novel antimicrobial agents to combat multidrug-resistant (MDR) infections, particularly those caused by carbapenem-resistant organisms (CROs), identified as priority pathogens by the World Health Organization (WHO) [1-4].

Among the limited therapeutic options available for the management of MDR infections, ceftazidime-avibactam (CZA) has gained significant attention as a promising treatment modality. CZA, a novel β-lactam/β-lactamase inhibitor combination antibiotic, has demonstrated notable efficacy against extended-spectrum β-lactamase (ESBL) producing Gram-negative bacilli (GNBs) and certain carbapenemase-producing organisms, including those belonging the *Klebsiella pneumoniae* carbapenemase (KPC) and OXA-48-like families. Despite its efficacy, CZA's utility is limited against metallo-β-lactamase (MBL)-producing GNBs [2,3].

While CZA has already been approved by the US Food and Drug Administration (USFDA) for specific conditions, including complicated intra-abdominal and urinary tract infections, its potential in treating bloodstream infections (BSIs) is gaining recognition. However, in resource-limited settings like India, where the prevalence of MDR infections is high, access to CZA remains constrained. Moreover, the lack of antimicrobial susceptibility testing (AST) data for CZA further complicates the rationale for its use [3-5]. Consequently, to optimize therapeutic outcomes and mitigate the emergence of AMR, AST-guided utilization of CZA is imperative, particularly in managing "difficult-to-treat" BSIs caused by MDROs. In light of this, this study explores the role of CZA as a therapeutic option for MDR gram-negative BSIs, emphasizing the importance of AST-guided treatment strategies in resource-limited healthcare settings.

## Materials and methods

Study design and setting

The study design entailed a laboratory-based prospective approach, spanning one year from April 2022 to March 2023. Subject selection was based on a convenient sampling technique. The study was conducted in the Department of Microbiology, after obtaining approval from the Institutional Ethical Committee (IEC), Jagadguru Sri Shivaratreeswara (JSS) Medical College, JSS Academy of Higher Education and Research, Mysuru (ref no: JSS/MC/PG/46/2022-23). Data collection involved retrieving clinical and sociodemographic information from the Hospital Information System, as well as samples submitted to the laboratory for testing and culture. The primary objective of the study was to ascertain the antimicrobial susceptibility profile of multidrug-resistant Gram-negative bacteria (MDR-GNB) in relation to CZA. We carried out a laboratory-based prospective investigation, and clinical and demographic data were retrieved from the Hospital Information System for subsequent analysis.

Inclusion and exclusion criteria

The inclusion criteria encompassed blood culture isolates identified as MDR Enterobacterales and MDR *Pseudomonas* species. The exclusion criteria encompassed organisms belonging to *Acinetobacter *species and other non-Enterobacterales, as well as isolates repetitively obtained from the same patient.

Lab techniques

Adhering to established laboratory procedures, positively flagged blood cultures via BACT/ALERT 3D (Biomerieux, Marcy l'Etoile, France) were processed. Identification and antimicrobial susceptibility testing were conducted using the VITEK-2 system (BioMerieux). CZA susceptibility testing with a concentration of 30/20 ug (HiMedia, Mumbai, India) was executed through the Kirby-Bauer disk diffusion method and minimum inhibitory concentration (MIC) testing via Epsilometer gradient diffusion test (E-test) using CZA E-strip (HiMedia) [6]. 

Kirby-Bauer Disk Diffusion Method for CZA

Briefly, the test organism was cultivated in broth for 18-24 hours before being contrasted to the 0.5 McFarland Turbidity standard. Mueller-Hinton agar was used to lawn culture the test isolates because it does not block sulphonamides and gives consistency with the medium's composition and pH. The lawn cultured plates were dried for about five minutes for the media to absorb the inoculum. Later, the forceps were sterilized and the antibiotic discs were picked up and placed at a distance of 24 mm each. The plates were incubated upside down for 24 hours at 37 °C. After 24 hours of incubation, a metric ruler was used to measure the zone of inhibition including the diameter of the disc in the measurement. The results were compared with the Clinical and Laboratory Standards Institute (CLSI) guidelines to report the test results. The results were reported as susceptible (S), intermediate (I), or resistant (R). A zone size of ≥21 was considered as S and a zone size of ≤ 20 was interpreted as R.

Epsilometer Test-CZA

E-test is a test for antimicrobial resistance utilizing an "exponential gradient" method. The E-test was developed to provide a precise evaluation of the bacteria's susceptibility to antibiotics. This method of quantification takes into account both antimicrobial dilution and antibacterial medium diffusion. The device comprises an exponential gradient of antibiotic concentrations that runs constantly over a plastic test strip. After 48 hours of incubation, an inhibition zone in the shape of a drop intersects the graded test strip at the inhibitory concentration (IC) of the antibiotic. In brief, an inoculum of 0.5 Mcfarland solution was prepared and a lawn culture of inoculum was performed on Mueller-Hinton agar plates. After drying the plates for around five minutes, the e-strip was placed on Mueller-Hinton agar plates and pressed with an applicator stick. The plates were incubated at 37 °C for 18-24 hours. 

Results and interpretations

MIC was read at the point where the ellipse intersects the scale. MIC values between two two-fold dilutions were always rounded up to the highest value. MIC values of the bacteria were interpreted as S or R by comparing the breakpoint values of each antibiotic based on the criteria recommended by CLSI. An MIC of ≤8/4 was considered as S and an MIC of ≥16/4 was considered as R.

## Results

During the study period, a total of 376 positive blood cultures were identified in the Department of Microbiology. Of these, 147 (42.4%) were found to be MDR Gram-negative organisms. Due to resource constraints and in alignment with the focus of postgraduate research, a subset of 100 MDR GNB samples was randomly selected for further analysis. The distribution of various MDR GNB organisms is detailed in Table 1, with *Klebsiella pneumoniae (K. pneumoniae)* comprising the majority (78, 78%), followed by *Escherichia coli (E. coli) *(10, 10%) and *Pseudomonas aeruginosa (P. aeruginosa) *(8, 8%). Carbapenems such as imipenem, ertapenem, meropenem, and doripenem were included in the study. Those isolates that were resistant (as per Vitek 2 susceptibility results) to any one of the specified carbapenems were considered carbapenem-resistant organisms (CROs). Among 92 MDR Enterobacterales, 69 (75%) were carbapenem-resistant organisms and 23 were (25%) carbapenem-susceptible.

**Table 1:** Distribution of MDR-GNB and overall susceptibility of CZA among these isolates CLSI: Clinical and Laboratory Standards Institute; CZA: ceftazidime-avibactam; DD: disk diffusion; E-test: Epsilometer test; MDR-GNB: multidrug-resistant Gram-negative bacteria; R: resistant; S: susceptible

Organisms (%)	CZA disk diffusion (mm)		CZA E-test (µg/mL)		Overall CZA susceptibility (DD + E-test), n (%)
	CLSI - 2022-23 breakpoints				
	S (≥21 mm), n (%)	R (≤20 mm), n (%)	S (≤8 µg/mL), n (%)	R (≥16 µg/mL), n (%)	
*E. coli* (10)	-	2 (20%)	5 (50%)	3 (30%)	5 (50%)
*K. pneumoniae* (78)	16 (20.5%)	28 (35.8%)	20 (25.6%)	14 (17.9%)	36 (46%)
*S. mercescens* (4)	3 (75%)	-	1 (25%)	0	4 (100%)
*P. aeruginosa* (8)	-	8 (100%)	0	0	0
Total	19 (19%)	38 (38%)	26 (26%)	17 (17%)	45 (45%)

Following the disk diffusion test, of 100 isolates, 43 (43%) exhibited zone sizes ranging between 17-20 mm and 22-25 mm, necessitating further testing through a MIC determined using the E-test, as per the study protocol. The test results of disc diffusion and E-test (DD + E-test) were assessed as per CLSI 2022/2023 guidelines. It was noted that 45% of the test isolates were susceptible to CZA (Table 1). CZA susceptibility was noted in 46 (46%) of *K. pneumoniae *and 50 (50%) among *E. coli*. Also, it is noteworthy that all eight (100%) of *P. aeruginosa* isolates were resistant to CZA.

When comparing CLSI-extrapolated breakpoints (CEB) with study-extrapolated breakpoints (SEB), using the E-test results as the gold standard, we found three major errors (two in *K. pneumoniae* and one in *E. coli*), as illustrated in Table 2.

**Table 2:** A scatter chart comparing the results of the CZA E-test and disk diffusion ^*^Major errors CZA: ceftazidime-avibactam; E-test: Epsilometer test; MIC: minimum inhibitory concentration

E-test MIC (μg/ml)	CZA-disk diffusion zone diameter (mm)											
	16	17	18	19	20	21	22	23	24	25	26	27
0.125	-	-	-	-	-	-	-	1 (2.3%)	-	-	-	-
0.38	-	-	-	-	-	-	-	-	-	2 (4.6%)	-	-
4	-		1 (2.3%)	-	-	-	1 (2.3%)	-	2 (4.6%)	-	-	-
6	-	-	-	-	1 (2.3%)	-	-	-	-	-	-	-
8	-	-	-	-	-	6 (13.9%)	4 (9.3%)	4 (9.3%)	-	3 (6.9%)	-	-
12	-	-	-	-	-	-	-	-	-	-	-	-
16	-	10 (23%)	2 (4.6%)	-	1 (2.3%)	-	-	1 (2.3%)	-	-	-	-
24	-	1 (2.3%)	-	-	-	-	-	-	-	-	-	-
48	-	1 (2.3%)	2^*^ (4.6%)	-	-	-	-	-	-	-	-	-
No. of evaluable results (n=43)	0	12 (27.6%)	5 (11.5%)	0	2 (4.6%)	6 (13.9%)	5 (11.6%)	6 (13.9%)	2 (4.6%)	5 (11.5%)	0	0

In the context of three major errors noted with CBE and SBE, we further examined the diagnostic performance of disk diffusion, using E-test results as the gold standard. The results of this analysis are presented in Table 3.

**Table 3:** Disk diffusion test performance vs. gold standard E-test CI: confidence interval; E-test: Epsilometer test

Parameters	Value	95% Cl
Sensitivity	94.12%	71.31–99.85%
Specificity	84.62%	65.13–95.64%
Accuracy	88.37%	74.92–96.11%

The majority (92, 92%) of BSIs caused by MDROs were associated with Enterobacterales, with *K. pneumoniae* (73, 73%) and *E. coli *(10, 10%) being the most prevalent species, followed by *P. aeruginosa* ​​​​​(8, 8%). A notable proportion of *K. pneumoniae *(42, 60.5%) were identified as CROs. An analysis of CZA's susceptibility profile for CROs and CSOs revealed a significant shift. The susceptibility to CZA among 81 CRO isolates was 34 (41.98%) and it was 8 (42.11%) among 19 CSE isolates. Notably, CR *K. pneumoniae* (30, 37.04%) exhibited higher susceptibility to CZA compared to CR *P. aeruginosa* (0%) and CR *E. coli* ​​​​​(4, 4.94%). This suggests that CZA may be a preferable treatment for BSIs caused by CR *K. pneumoniae* over other CROs.

To explore CZA susceptibility distribution, we looked into the susceptibility pattern of CZA among carbapenem-resistant Enterobacterales (CRE) and carbapenem-susceptible Enterobacterales (CSE). Our results showed a 42% susceptibility rate among CRE and 70% among CSE (Table 4).

**Table 4:** Organism-wise CZA susceptibility among CRE and CSE CRE: carbapenem-resistant Enterobacterales; CSE: carbapenem-susceptible Enterobacterales; CZA: ceftazidime-avibactam

Organisms	CZA susceptibility among CRE, % (n/N)	CZA susceptibility among CSE, % (n/N)
*E. coli* (n=10)	33 (1/3)	57 (4/7)
*K. pneumoniae* (n=78)	42 (28/66)	66 (8/12)
*S. marcescens* (n=4)	0 (0/0)	100 (4/4)
Total (n=92)	42 (29/69)	70 (16/23)

## Discussion

In the era of antimicrobial resistance with few cards to play, clinical microbiology laboratories need to have a reliable testing method for available treatment options. It is also noteworthy that there has been an increase in the rates of “difficult to treat” BSIs due to MDROs globally, which are resistant to carbapenem as well as various other β-lactam groups of antimicrobials, leaving very limited therapeutic options. Given this problem, it is very crucial to be aware of the susceptibility patterns of newly available agents and their efficacy in treating MDR infections [7-10]. Hence, in the present study, we evaluated the efficacy of the most commonly used method, i.e. disk diffusion to predict the susceptibility of CZA, and these results were compared with the gold standard E-test.

Of the 376 positive blood cultures, 147 (39.09%) were found to be MDR Gram-negative organisms, and 100 randomly picked isolates were chosen for further analysis by CZA susceptibility testing; 45 (45%) of the isolates were found to be susceptible to CZA by disc diffusion. Among these isolates, *E. coli* showed the highest number (5, 50%) of susceptible results, followed by *K. pneumoniae* ​​​​​​(35, 46%), and *Serratia marcescens* (*S*. *marcescens)* (4, 100%). In our study, we also found that all eight MDR *P. aeruginosa* (100%) isolates were resistant to CZA. Similar findings were noted by Ketan et al. [11], and overall CZA susceptibility was found to be 30.6% in their study, of which *E. coli* accounted for 27.5%, *K. pneumoniae* 37.3%, and *P. aeruginosa* 3.2%. All these results align with our findings, except for those of *P. aeruginosa,* which was 0% in our study.

In a multicentric study by Yamunadevi et al. [10], involving *E. coli* (n=458) and *K. pneumoniae* (n=455) isolates obtained from nine centers across India, the overall susceptibility to CZA was observed to be 72% among *K. pneumoniae *isolates and 87% among *E. coli* isolates. These results were in concordance with a study conducted at the Chughtai Institute of Pathology, Lahore, Pakistan [12], which reported an overall resistance of CZA of 77% among CRE isolates and 80% among CRPA isolates. As a novel approach, in which diffusion results were compared to CLSI-extrapolated breakpoints and study-extrapolated breakpoints using the E-test as the gold standard, our study reported three major errors (two in *K. pneumoniae* and one in *E. coli*). These findings have to be validated using a larger number of isolates. 

A study conducted by Qi Wang et al. [13] showed a very major error of 1.5%, a major error of 2.5% compared to the broth microdilution test, and another study conducted by Jingjia et al. [14] reported very major errors of 4.5%, which contrasts with our study. The overall sensitivity and specificity of the assay were found to be 94.12% and 84.62% respectively with an accuracy of 88.37%. Furthermore, CZA susceptibility testing results were compared between CRE and CSE and it was found that CZA is susceptible in 42% of CRE isolates and 70% of CSE isolates. Similar results were reported by Ketan et al.'s study [9], in which about 30.6% of MDROs were susceptible to CZA, whereas the CZA susceptibility among CRO and CSO was 21.6% and 92.7%, respectively.

Limitations of the study

We recommend further studies in which greater sample sizes are experimentally verified. According to our outcomes, CZA was most effective against Enterobacterales when the zone diameters were 20-21 mm. To prevent false-susceptible or false-resistant outcomes, broth microdilution testing should be performed, as per the recommendation of CLSI. However, our findings agreed with those from earlier studies. Another major limitation is that the clinical outcomes among the patients with CZA resistance were not determined. A few other limitations also need to be taken into account; the inoculum effect and the measurement of the inhibitory zone are two factors that affect the susceptibility testing of CZA, especially regarding isolates with CZA zones of 20-21 mm, because this zone range is on the border between the resistant and susceptible categories and might be the cause of the error. 

For antimicrobial therapy to be oriented towards monotherapy or combinational therapy, more testing is required to show the expression of certain enzymes. The most significant limitation of our study is that we did not conduct laboratory testing to find enzymes such as NDM, MBL, and OXA, which would have helped justify the use of antibiotics. A further limitation of the study is that the clinical response in the patients who started taking CZA was not evaluated.

## Conclusions

Our study sheds light on the critical challenge posed by MDR Gram-negative bloodstream infections, emphasizing the need for effective treatment options in the era of antimicrobial resistance. CZA demonstrates varying susceptibility patterns across different organisms, with notable effectiveness against certain isolates but limited efficacy against others, notably *P. aeruginosa*. Discrepancies between disk diffusion and gold standard testing underscore the need for rigorous validation and standardization in susceptibility-testing protocols. Further research with higher sample sizes is imperative to corroborate our findings and guide clinical decision-making in combating MDR infections effectively.
